# Synthesis and crystallographic characterization of [2,2-bis­(η^5^-penta­methyl­cyclo­penta­dien­yl)-3,4-bis(tri­methyl­sil­yl)-2-zircona­furan-5-one-κ*O* ^5^]triisobutyl­aluminium

**DOI:** 10.1107/S2056989018004759

**Published:** 2018-03-27

**Authors:** Vladimir V. Burlakov, Vyacheslav S. Bogdanov, Perdita Arndt, Anke Spannenberg, Uwe Rosenthal, Torsten Beweries, Vladimir B. Shur

**Affiliations:** a A. N. Nesmeyanov Institute of Organoelement Compounds, Russian Academy of, Sciences, Vavilov St. 28, 119991, Moscow, Russian Federation; b Leibniz-Institut für Katalyse e. V. an der Universität Rostock, Albert-Einstein-Strasse 29a, 18059 Rostock, Germany

**Keywords:** crystal structure, zirconium metallocene, triiso­butyl­aluminium, zwitterionic enolate complex

## Abstract

The crystal structure of the title zwitterionic zirconocene complex containing a furan­one unit, [AlZr(C_10_H_15_)_2_(C_4_H_9_)_3_(C_9_H_18_O_2_Si_2_)], is reported. On reacting a zircona­furan­one with two equivalents of HAl(*i*-Bu)_2_, disproportionation of the Lewis acid results in the formation of a triiso­butyl­aluminium fragment, Al(*i*-Bu)_3_, which coordinates to the exocyclic carbonyl O atom of the zircona­furan­one ring. Single-crystal X-ray diffraction reveals that the zircona­furan­one ring remains intact with coordination of the aluminium to the exocyclic O atom. One of the *i*-butyl groups is disordered over two sets of sites, with an occupancy ratio of 0.731 (3):0.269 (3).

## Abstract 2

The crystal structure is reported of a zwitterionic zirconocene complex containing a furan­one unit, namely [2,2-bis­(η^5^-penta­methyl­cyclo­penta­dien­yl)-3,4-bis­(tri­methyl­sil­yl)-2-zircona­furan-5-one-κ*O* ^5^]triiso­butyl­aluminium, in which the exocyclic carbonyl oxygen is coordinated to the aluminium atom of an Al(*i*-Bu)_3_ group.

## Chemical context   

Metallocene complexes of early transition metals can be activated by strong Lewis acids for many catalytic purposes. Reactions of group 4 metallocene complexes with Lewis acids such as HAl(*i*-Bu)_2_, Al(*i*-Bu)_3_ and also B(C_6_F_5_)_3_ are therefore of great inter­est and have been studied intensively (Brintzinger *et al.*, 1995 ▸). It has been reported previously that titana- and zirconacycles react readily with Al(*i*-Bu)_3_/HAl(*i*-Bu)_2_ to give either heterobimetallic complexes with inter­esting structural features (Erker *et al.*, 1992 ▸; Arndt *et al.*, 2001 ▸) or zwitterionic binuclear compounds (Erker *et al.*, 1992 ▸; Burlakov *et al.*, 2004 ▸, 2006 ▸, 2011 ▸). The latter demonstrated remarkable catalytic activity in the ROP of ∊-caprolactone (Arndt *et al.*, 1996 ▸; Arndt *et al.*, 1997 ▸). The structure of a zwitterionic zirconocene ester enolate complex and a tantalactone, each coordinated to Al(C_6_F_5_)_3_ units, were reported recently (Tsurugi *et al.*, 2006 ▸). The role of the zirconocene complex as an inter­mediate in the isospecific polymerization of methacrylates has been discussed (Zr: Bolig & Chen, 2004 ▸; Ta: Tsurugi *et al.*, 2006 ▸). Recently, we found that the reaction of a zirconadi­hydro­furan with HAl(*i*-Bu)_2_ gave a 1:1 complex where, in addition to the coordination of the aluminium atom to the oxygen of the intact furan ring, a Zr–H–Al bridge was obtained. This compound also behaves as an active catalyst in the ROP of ∊-caprolactone (Burlakov *et al.*, 2017 ▸). In addition, a zwitterionic hafnocene furan­one–B(C_6_F_5_)_3_ adduct has been synthesized and structurally characterized (Beweries *et al.*, 2009 ▸). We were therefore inter­ested in the reactivity of the zirconafuran­one **1**, whose crystal structure has been reported (Pellny *et al.*, 1999 ▸), towards HAl(*i*-Bu)_2_.
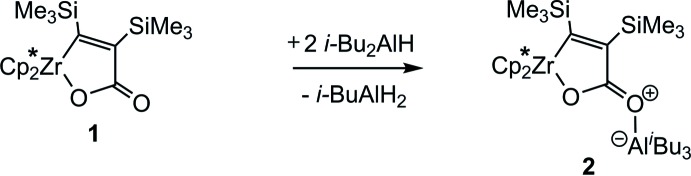


In the present work, the zirconafuran­one **1** reacts with two equivalents of HAl(*i*-Bu)_2_, and a disproportionation of the Lewis acid gives a triiso­butyl­aluminium fragment, leading to the formation of the zwitterionic title compound **2** by coordination of Al(*i*-Bu)_3_ to the exocyclic carbonyl oxygen of the zircona­furan­one ring (see Scheme[Chem scheme1]).

## Structural commentary   

**Figure 1:**
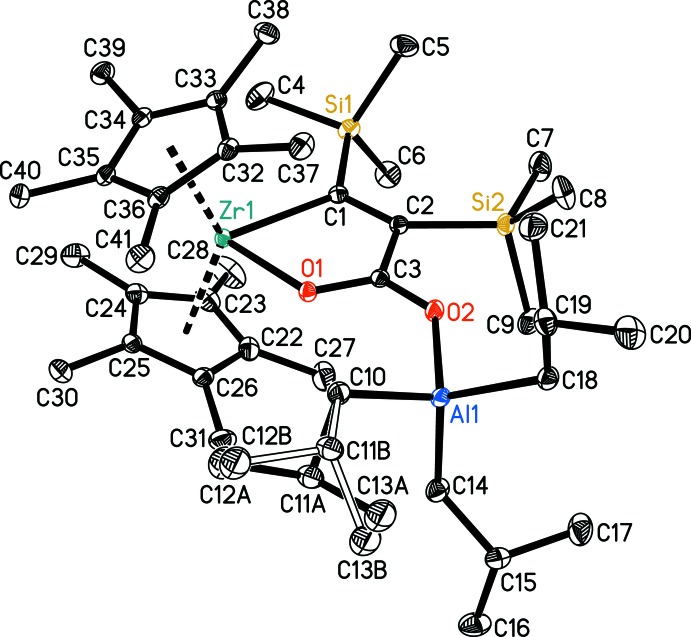
The mol­ecular structure of the title complex **2** with the atom labelling. Displacement ellipsoids correspond to the 30% probability level. H atoms have been omitted for clarity. The minor disorder component is indicated by open bonds.

**Figure 2:**
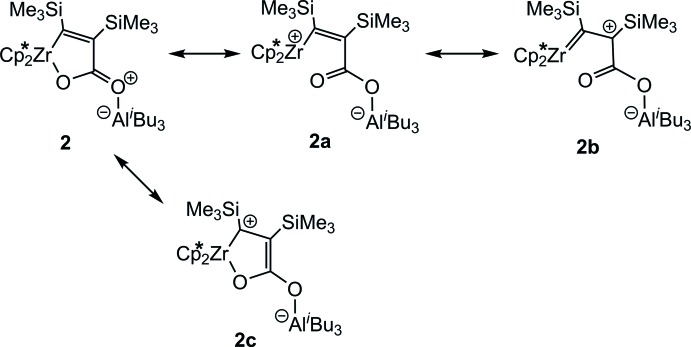
Possible resonance structures of complex **2**.

The mol­ecular structure of **2** (Fig. 1 ▸) shows a bent zirconocene unit, together with a planar five-membered metallacycle (the intact zircona­furan­one) with an aluminium atom of the *i*-Bu_3_Al group coordinated to the exocyclic oxygen atom. The values of the Al1—O2 distance [1.9016 (10) Å] and the Al1—O2—C3 angle [134.62 (9)°] are as expected. As a result of the complexation of the organoaluminium unit in **2**, the C3—O2 bond is essentially elongated compared to that in the starting complex **1** [**1**: 1.222 (6), **2**: 1.2605 (15) Å)] whereas the C3—O1 bond is shortened [**1**: 1.326 (6), **2**: 1.2819 (15) Å]. This shortening is accompanied by an elongation of the Zr1—O1 distance [**1**: 2.048 (4), **2**: 2.0891 (9) Å] and a slight decrease in the C2—C3 distance [**1**: 1.524 (7), **2**: 1.5055 (18) Å]. All these bond lengths lie in the expected ranges and similar values have been reported for a hafnocene complex coordinated with B(C_6_F_5_)_3_ (Beweries *et al.*, 2009 ▸), and for zwitterionic adducts of the Lewis acid Al(C_6_F_5_)_3_ to either a zirconocene enolate (Bolig *et al.*, 2004 ▸) or a tantalalactone (Tsurugi *et al.*, 2006 ▸). These effects can be explained by a contribution of the resonance forms **2a**–**2c** to the electronic structure of complex **2** (Fig. 2 ▸).

The zircona­furan­one metallacycle in **2** retains its virtually planar structure. The endocyclic C1—Zr1—O1 bond angle [74.44 (4)°] is close to that in the starting complex **1** [75.5 (2)°]. The Al atom deviates from the zircona­furan­one plane by 0.21 Å.

## Supra­molecular features   

**Figure 3:**
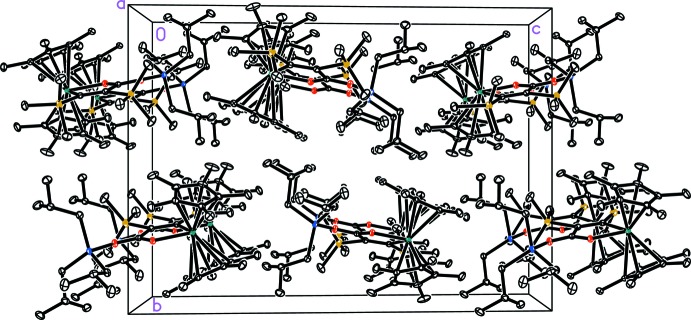
Packing diagram for **2** viewed along the *a* axis. Displacement ellipsoids correspond to the 30% probability level. H atoms and lower occupancy sites have been omitted for clarity.

For the title complex **2** no significant supra­molecular features are observed. The crystal packing appears to be dominated by van der Waals inter­actions (Fig. 3 ▸).

## Synthesis and crystallisation   

All operations were carried out under argon with standard Schlenk techniques or in a glovebox. The starting zirconona­furan­one **1** was prepared according to a method previously described in the literature (Pellny *et al.*, 1999 ▸).

A commercial 1 *M* solution of ^*i*^Bu_2_AlH in cyclo­hexane was purchased from Sigma Aldrich and used as received. Solvents were purified by conventional methods and were distilled twice over metallic sodium (toluene, *n*-hexa­ne) under Ar prior to use. The ^1^H and ^13^C NMR spectra were recorded on Bruker AMX-400 and AV-400 spectrometers. The IR spectra were recorded on a Nicolet Magna IR-750 FTIR spectrometer. The mass spectra were measured using a MAT 95-XP instrument.

**Synthesis of 2:** To a solution of **1** (0.216 g, 0.38 mmol) in 7–8 mL of toluene were added 0.8 mL of a 1 *M* solution of ^*i*^Bu_2_AlH (0.8 mmol) in cyclo­hexane. The resulting mixture was stirred for several minutes and then allowed to stand under Ar at room temperature. After one day, the resulting yellow solution was evaporated under vacuum to give an oily yellow residue. Then, *n*-hexane (1.0–1.5 mL) was added and the solution obtained allowed to stand overnight at room temperature. The following day, the precipitated fine crystalline orange complex **2** was separated from the mother liquor by deca­nting, washed with cold *n*-hexane and dried in vacuum. Yield of **2**: 0.221 g (74%). A recrystallization of the complex from *n*-hexane gave 0.114 g of red–orange crystals of **2** suitable for an X-ray diffraction study. M.p. 434–436 K (dec.) under Ar. C_41_H_75_AlO_2_Si_2_Zr (774.41): calculated C 63.59, H 9.76; found C 63.31, H 9.63%. ^1^H NMR (C_6_D_6_, 295K, δ, ppm): −0.43 (*s*, *br*, 3H, *α*-SiMe), 0.28 (*s*, *br*, 6H, *α*-SiMe_2_), 0.51 (*d*, ^3^ *J* = 7.0 Hz, 6H, CH_2_); 0.55 (*s*, 9H, *β*-SiMe_3_), 1.48 (*d*, ^3^ *J* = 6.6 Hz, 18H, CH_3_); 1.66 (*s*, 30H, Cp*); 2.46 (*m*, 3H, CH). ^13^C NMR (C_6_D_6_, 295K, δ, ppm): 3.4 (*β*-SiMe_3_); 11.9 (C_5_Me_5_); 25.8 (CH_2_); 27.7 (CH); 29.5 (CH_3_); 124.4 (C_5_Me_5_); 169.5 (C=O); 172.8 (*β*-C); at 295 K the signals of *α*-C, and *α*-SiMe_3_ are not observed. IR (ATR, cm^−1^): ν_s_CO_2_, 1340; ν_as_CO_2_, 1520. MS (70 eV, *m*/*z*): 675 [*M* − C_2_SiMe_3_]^+^, 574 [*M* − ^*i*^Bu_3_Al]^+^, 360 [Cp*_2_Zr]^+^.

## Refinement   

Crystal data, data collection and structure refinement details are summarized in Table 1 ▸. All H atoms were placed in idealized positions and refined using a riding model: C—H = 0.98–1.00 Å with *U*_iso_(H) = 1.5*U*_eq_(C-methyl) and 1.2*U*_eq_(C) for other H atoms. One of the *i*-butyl groups was found to be disordered over two sets of sites (C11*A*, C12*A*, C13*A*/C11*B*, C12*B*, C13*B*) with an occupancy ratio of 0.731 (3):0.269 (3). The EADP instruction was used during modelling of this group. The DFIX instruction was used for restraining the distance C11*B*–C13*B*.

## Supplementary Material

Crystal structure: contains datablock(s) I, New_Global_Publ_Block. DOI: 10.1107/S2056989018004759/cq2023sup1.cif

Structure factors: contains datablock(s) I. DOI: 10.1107/S2056989018004759/cq2023Isup2.hkl

CCDC reference: 1831775

Additional supporting information:  crystallographic information; 3D view; checkCIF report

## Figures and Tables

**Table 1:** Experimental details

Crystal data	
Chemical formula	[AlZr(C_10_H_15_)_2_(C_4_H_9_)_3_(C_9_H_18_O_2_Si_2_)]
*M*_r_	774.39
Crystal system, space group	Monoclinic, *P*2_1_/*n*
Temperature (K)	150
*a*, *b*, *c* (Å)	11.5404 (2), 16.5073 (3), 22.9519 (4)
β (°)	95.0206 (9)
*V* (Å^3^)	4355.58 (13)
*Z*	4
Radiation type	Mo *K*α
μ (mm^−1^)	0.36
Crystal size (mm)	0.53 × 0.32 × 0.19

Data collection	
Diffractometer	Bruker APEXII CCD
Absorption correction	Multi-scan (*SADABS*; Bruker, 2011 ▸)
*T*_min_, *T*_max_	0.671, 0.746
No. of measured, independent and observed [*I* > 2σ(*I*)] reflections	97067, 10803, 9409
*R*_int_	0.034
(sin θ/λ)_max_ (Å^−1^)	0.667

Refinement	
*R*[*F* ^2^ > 2σ(*F* ^2^)], *wR*(*F* ^2^), *S*	0.026, 0.068, 1.04
No. of reflections	10803
No. of parameters	448
No. of restraints	1
H-atom treatment	H-atom parameters constrained
Δρ_max_, Δρ_min_ (e Å^−3^)	0.37, −0.29

Computer programs: *APEX2* and, *SAINT* (Bruker, 2011 ▸), *SHELXS97* (Sheldrick, 2008 ▸), *SHELXL2014* (Sheldrick, 2015 ▸), *XP* in *SHELXTL* (Sheldrick, 2008 ▸) and *publCIF* (Westrip, 2010 ▸).

## supplementary crystallographic information

### Crystal data

**Table:**

[AlZr(C_10_H_15_)_2_(C_4_H_9_)_3_(C_9_H_18_O_2_Si_2_)]	*F*(000) = 1672
*M**_r_* = 774.39	*D*_x_ = 1.181 Mg m^−^^3^
Monoclinic, *P*2_1_/*n*	Mo *K*α radiation, λ = 0.71073 Å
*a* = 11.5404 (2) Å	Cell parameters from 9039 reflections
*b* = 16.5073 (3) Å	θ = 2.3–28.6°
*c* = 22.9519 (4) Å	µ = 0.36 mm^−^^1^
β = 95.0206 (9)°	*T* = 150 K
*V* = 4355.58 (13) Å^3^	Prism, orange
*Z* = 4	0.53 × 0.32 × 0.19 mm

### Data collection

**Table:**

Bruker APEXII CCD diffractometer	10803 independent reflections
Radiation source: fine-focus sealed tube	9409 reflections with *I* > 2σ(*I*)
Curved graphite monochromator	*R*_int_ = 0.034
φ and ω scans	θ_max_ = 28.3°, θ_min_ = 1.5°
Absorption correction: multi-scan (SADABS; Bruker, 2011)	*h* = −15→15
*T*_min_ = 0.671, *T*_max_ = 0.746	*k* = −22→21
97067 measured reflections	*l* = −30→30

### Refinement

**Table:**

Refinement on *F*^2^	1 restraint
Least-squares matrix: full	Hydrogen site location: inferred from neighbouring sites
*R*[*F*^2^ > 2σ(*F*^2^)] = 0.026	H-atom parameters constrained
*wR*(*F*^2^) = 0.068	*w* = 1/[σ^2^(*F*_o_^2^) + (0.0308*P*)^2^ + 2.1095*P*] where *P* = (*F*_o_^2^ + 2*F*_c_^2^)/3
*S* = 1.04	(Δ/σ)_max_ = 0.003
10803 reflections	Δρ_max_ = 0.37 e Å^−^^3^
448 parameters	Δρ_min_ = −0.29 e Å^−^^3^

### Special details

**Table:**

Geometry. All esds (except the esd in the dihedral angle between two l.s. planes) are estimated using the full covariance matrix. The cell esds are taken into account individually in the estimation of esds in distances, angles and torsion angles; correlations between esds in cell parameters are only used when they are defined by crystal symmetry. An approximate (isotropic) treatment of cell esds is used for estimating esds involving l.s. planes.

### Fractional atomic coordinates and isotropic or equivalent isotropic displacement parameters (Å^2^)

**Table:**

	*x*	*y*	*z*	*U*_iso_*/*U*_eq_	Occ. (<1)
Al1	0.31511 (3)	0.78416 (2)	−0.06571 (2)	0.01847 (8)	
C1	0.07740 (11)	0.70811 (8)	0.11621 (6)	0.0180 (2)	
C2	0.07292 (11)	0.72120 (8)	0.05721 (6)	0.0180 (2)	
C3	0.18627 (11)	0.74781 (8)	0.03551 (5)	0.0170 (2)	
C4	−0.02054 (18)	0.67250 (11)	0.23547 (7)	0.0410 (4)	
H4A	−0.0456	0.7228	0.2532	0.061*	
H4B	0.0635	0.6657	0.2444	0.061*	
H4C	−0.0614	0.6265	0.2513	0.061*	
C5	−0.17679 (13)	0.75327 (11)	0.14051 (8)	0.0376 (4)	
H5A	−0.2495	0.7248	0.1283	0.056*	
H5B	−0.1582	0.7909	0.1097	0.056*	
H5C	−0.1858	0.7835	0.1766	0.056*	
C6	−0.10857 (15)	0.57277 (10)	0.13180 (8)	0.0365 (4)	
H6A	−0.0856	0.5343	0.1632	0.055*	
H6B	−0.0743	0.5563	0.0961	0.055*	
H6C	−0.1935	0.5734	0.1246	0.055*	
C7	−0.19217 (14)	0.67444 (12)	0.00642 (8)	0.0399 (4)	
H7A	−0.2274	0.7157	0.0300	0.060*	
H7B	−0.1882	0.6228	0.0276	0.060*	
H7C	−0.2395	0.6678	−0.0309	0.060*	
C8	−0.06282 (15)	0.80310 (12)	−0.05034 (8)	0.0401 (4)	
H8A	0.0047	0.8125	−0.0726	0.060*	
H8B	−0.0710	0.8484	−0.0234	0.060*	
H8C	−0.1331	0.7989	−0.0774	0.060*	
C9	0.01225 (16)	0.62376 (12)	−0.05374 (8)	0.0412 (4)	
H9A	−0.0447	0.6135	−0.0871	0.062*	
H9B	0.0232	0.5744	−0.0302	0.062*	
H9C	0.0866	0.6398	−0.0679	0.062*	
C10	0.43851 (12)	0.84505 (9)	−0.01800 (6)	0.0227 (3)	
H10A	0.4138	0.9024	−0.0165	0.027*	0.731 (3)
H10B	0.4424	0.8238	0.0225	0.027*	0.731 (3)
H10C	0.3991	0.8872	0.0038	0.027*	0.269 (3)
H10D	0.4749	0.8069	0.0114	0.027*	0.269 (3)
C14	0.36565 (14)	0.67117 (9)	−0.08337 (6)	0.0280 (3)	
H14A	0.4402	0.6615	−0.0596	0.034*	
H14B	0.3079	0.6338	−0.0685	0.034*	
C15	0.38273 (13)	0.64510 (9)	−0.14575 (6)	0.0258 (3)	
H15	0.4333	0.6862	−0.1630	0.031*	
C16	0.44275 (17)	0.56268 (10)	−0.14793 (8)	0.0404 (4)	
H16A	0.5201	0.5657	−0.1267	0.061*	
H16B	0.4505	0.5478	−0.1888	0.061*	
H16C	0.3962	0.5217	−0.1297	0.061*	
C17	0.26749 (17)	0.64230 (11)	−0.18318 (8)	0.0434 (4)	
H17A	0.2164	0.6022	−0.1671	0.065*	
H17B	0.2812	0.6271	−0.2233	0.065*	
H17C	0.2305	0.6958	−0.1833	0.065*	
C18	0.24126 (12)	0.84926 (8)	−0.13239 (6)	0.0229 (3)	
H18A	0.2814	0.8363	−0.1676	0.028*	
H18B	0.1595	0.8313	−0.1400	0.028*	
C19	0.24145 (13)	0.94209 (9)	−0.12488 (6)	0.0266 (3)	
H19	0.3200	0.9581	−0.1066	0.032*	
C20	0.22088 (16)	0.98725 (10)	−0.18296 (8)	0.0378 (4)	
H20A	0.1437	0.9735	−0.2016	0.057*	
H20B	0.2804	0.9714	−0.2087	0.057*	
H20C	0.2254	1.0458	−0.1758	0.057*	
C21	0.15199 (15)	0.96891 (10)	−0.08371 (7)	0.0347 (3)	
H21A	0.1553	1.0279	−0.0791	0.052*	
H21B	0.1691	0.9430	−0.0455	0.052*	
H21C	0.0740	0.9530	−0.1001	0.052*	
C22	0.30471 (14)	0.58593 (9)	0.12621 (7)	0.0289 (3)	
C23	0.27096 (13)	0.57747 (9)	0.18382 (8)	0.0302 (3)	
C24	0.35679 (13)	0.61435 (9)	0.22298 (6)	0.0247 (3)	
C25	0.44621 (12)	0.64332 (8)	0.18925 (6)	0.0216 (3)	
C26	0.41274 (13)	0.62635 (9)	0.12985 (6)	0.0244 (3)	
C27	0.24605 (18)	0.54962 (11)	0.07126 (9)	0.0462 (5)	
H27A	0.2520	0.5872	0.0386	0.069*	
H27B	0.1639	0.5396	0.0765	0.069*	
H27C	0.2840	0.4984	0.0628	0.069*	
C28	0.17516 (17)	0.52383 (11)	0.20124 (11)	0.0537 (6)	
H28A	0.2047	0.4686	0.2074	0.081*	
H28B	0.1113	0.5237	0.1702	0.081*	
H28C	0.1468	0.5442	0.2375	0.081*	
C29	0.36174 (17)	0.60883 (11)	0.28868 (7)	0.0394 (4)	
H29A	0.2874	0.6267	0.3019	0.059*	
H29B	0.4244	0.6436	0.3060	0.059*	
H29C	0.3767	0.5526	0.3009	0.059*	
C30	0.56634 (13)	0.67020 (10)	0.21131 (7)	0.0319 (3)	
H30A	0.6228	0.6288	0.2022	0.048*	
H30B	0.5700	0.6781	0.2537	0.048*	
H30C	0.5846	0.7213	0.1924	0.048*	
C31	0.48657 (15)	0.64314 (11)	0.08034 (7)	0.0373 (4)	
H31A	0.5489	0.6028	0.0806	0.056*	
H31B	0.5206	0.6974	0.0850	0.056*	
H31C	0.4383	0.6402	0.0431	0.056*	
C32	0.24716 (12)	0.88143 (8)	0.15538 (6)	0.0211 (3)	
C33	0.17173 (12)	0.85625 (8)	0.19759 (6)	0.0207 (3)	
C34	0.24156 (12)	0.82389 (8)	0.24646 (6)	0.0211 (3)	
C35	0.35968 (12)	0.83082 (8)	0.23447 (6)	0.0213 (3)	
C36	0.36309 (12)	0.86491 (8)	0.17777 (6)	0.0218 (3)	
C37	0.20965 (15)	0.92403 (9)	0.09909 (6)	0.0298 (3)	
H37A	0.2009	0.9821	0.1066	0.045*	
H37B	0.1351	0.9018	0.0826	0.045*	
H37C	0.2685	0.9161	0.0713	0.045*	
C38	0.04497 (13)	0.87667 (9)	0.19567 (7)	0.0299 (3)	
H38A	0.0359	0.9324	0.2094	0.045*	
H38B	0.0064	0.8393	0.2210	0.045*	
H38C	0.0097	0.8717	0.1554	0.045*	
C39	0.20212 (14)	0.80374 (10)	0.30555 (6)	0.0303 (3)	
H39A	0.2394	0.7534	0.3200	0.045*	
H39B	0.1174	0.7969	0.3022	0.045*	
H39C	0.2238	0.8479	0.3329	0.045*	
C40	0.46210 (14)	0.82334 (10)	0.27918 (7)	0.0319 (3)	
H40A	0.5334	0.8174	0.2593	0.048*	
H40B	0.4521	0.7757	0.3037	0.048*	
H40C	0.4677	0.8720	0.3037	0.048*	
C41	0.47167 (14)	0.88740 (10)	0.14958 (7)	0.0329 (3)	
H41A	0.4536	0.8921	0.1072	0.049*	
H41B	0.5308	0.8454	0.1579	0.049*	
H41C	0.5012	0.9393	0.1653	0.049*	
O1	0.27625 (8)	0.75358 (6)	0.07220 (4)	0.01799 (18)	
O2	0.19180 (8)	0.76400 (6)	−0.01785 (4)	0.02080 (19)	
Si1	−0.05531 (3)	0.67729 (2)	0.15391 (2)	0.02370 (8)	
Si2	−0.04190 (3)	0.70704 (3)	−0.00788 (2)	0.02353 (8)	
Zr1	0.26628 (2)	0.72870 (2)	0.16087 (2)	0.01563 (4)	
C11A	0.56192 (17)	0.84307 (13)	−0.03814 (9)	0.0264 (4)	0.731 (3)
H11A	0.5889	0.7855	−0.0374	0.032*	0.731 (3)
C12A	0.6450 (4)	0.8917 (3)	0.00443 (16)	0.0431 (6)	0.731 (3)
H12A	0.6416	0.8706	0.0442	0.065*	0.731 (3)
H12B	0.7246	0.8867	−0.0070	0.065*	0.731 (3)
H12C	0.6219	0.9488	0.0033	0.065*	0.731 (3)
C13A	0.5658 (2)	0.8747 (2)	−0.10002 (11)	0.0431 (6)	0.731 (3)
H13A	0.5123	0.8432	−0.1267	0.065*	0.731 (3)
H13B	0.5424	0.9318	−0.1015	0.065*	0.731 (3)
H13C	0.6450	0.8697	−0.1118	0.065*	0.731 (3)
C11B	0.5373 (5)	0.8867 (4)	−0.0468 (2)	0.0264 (4)	0.269 (3)
H11B	0.5062	0.9379	−0.0656	0.032*	0.269 (3)
C12B	0.6443 (11)	0.9079 (8)	−0.0060 (5)	0.0431 (6)	0.269 (3)
H12D	0.6213	0.9422	0.0259	0.065*	0.269 (3)
H12E	0.6801	0.8580	0.0103	0.065*	0.269 (3)
H12F	0.7004	0.9371	−0.0279	0.065*	0.269 (3)
C13B	0.5838 (6)	0.8341 (5)	−0.0938 (3)	0.0431 (6)	0.269 (3)
H13D	0.5195	0.8178	−0.1221	0.065*	0.269 (3)
H13E	0.6411	0.8648	−0.1139	0.065*	0.269 (3)
H13F	0.6208	0.7857	−0.0758	0.065*	0.269 (3)

### Atomic displacement parameters (Å^2^)

**Table:**

	*U*^11^	*U*^22^	*U*^33^	*U*^12^	*U*^13^	*U*^23^
Al1	0.02091 (19)	0.02017 (19)	0.01445 (17)	0.00112 (15)	0.00230 (14)	0.00134 (14)
C1	0.0193 (6)	0.0154 (6)	0.0196 (6)	−0.0007 (5)	0.0029 (5)	−0.0006 (5)
C2	0.0171 (6)	0.0180 (6)	0.0189 (6)	−0.0006 (5)	0.0019 (5)	−0.0008 (5)
C3	0.0194 (6)	0.0146 (6)	0.0169 (6)	0.0018 (4)	0.0012 (4)	−0.0003 (4)
C4	0.0602 (11)	0.0401 (9)	0.0246 (8)	−0.0098 (8)	0.0148 (7)	0.0029 (7)
C5	0.0209 (7)	0.0409 (9)	0.0519 (10)	−0.0016 (6)	0.0084 (7)	−0.0104 (8)
C6	0.0341 (8)	0.0295 (8)	0.0458 (10)	−0.0116 (7)	0.0030 (7)	0.0004 (7)
C7	0.0252 (8)	0.0575 (11)	0.0361 (9)	−0.0119 (7)	−0.0030 (6)	0.0010 (8)
C8	0.0295 (8)	0.0482 (10)	0.0406 (9)	−0.0011 (7)	−0.0088 (7)	0.0158 (8)
C9	0.0397 (9)	0.0488 (11)	0.0341 (9)	−0.0034 (8)	−0.0029 (7)	−0.0188 (8)
C10	0.0219 (6)	0.0266 (7)	0.0199 (6)	−0.0007 (5)	0.0031 (5)	0.0011 (5)
C14	0.0378 (8)	0.0247 (7)	0.0214 (7)	0.0053 (6)	0.0025 (6)	0.0024 (5)
C15	0.0299 (7)	0.0225 (7)	0.0259 (7)	0.0017 (6)	0.0077 (6)	0.0007 (5)
C16	0.0498 (10)	0.0292 (8)	0.0440 (10)	0.0112 (7)	0.0136 (8)	−0.0027 (7)
C17	0.0529 (11)	0.0340 (9)	0.0401 (10)	0.0050 (8)	−0.0143 (8)	−0.0113 (7)
C18	0.0271 (7)	0.0240 (7)	0.0177 (6)	0.0020 (5)	0.0014 (5)	0.0020 (5)
C19	0.0278 (7)	0.0235 (7)	0.0274 (7)	0.0011 (6)	−0.0036 (6)	0.0038 (6)
C20	0.0455 (10)	0.0294 (8)	0.0386 (9)	0.0059 (7)	0.0036 (7)	0.0133 (7)
C21	0.0435 (9)	0.0278 (8)	0.0320 (8)	0.0049 (7)	0.0001 (7)	−0.0039 (6)
C22	0.0360 (8)	0.0162 (6)	0.0326 (8)	0.0070 (6)	−0.0085 (6)	−0.0028 (6)
C23	0.0294 (7)	0.0162 (6)	0.0445 (9)	0.0016 (6)	0.0008 (6)	0.0086 (6)
C24	0.0290 (7)	0.0209 (7)	0.0244 (7)	0.0067 (5)	0.0037 (5)	0.0080 (5)
C25	0.0231 (6)	0.0208 (6)	0.0205 (6)	0.0060 (5)	0.0003 (5)	0.0019 (5)
C26	0.0312 (7)	0.0215 (7)	0.0202 (6)	0.0105 (6)	0.0008 (5)	0.0005 (5)
C27	0.0544 (11)	0.0280 (8)	0.0517 (11)	0.0097 (8)	−0.0207 (9)	−0.0165 (8)
C28	0.0392 (10)	0.0283 (9)	0.0938 (17)	−0.0047 (8)	0.0073 (10)	0.0242 (10)
C29	0.0532 (10)	0.0405 (9)	0.0259 (8)	0.0163 (8)	0.0114 (7)	0.0143 (7)
C30	0.0247 (7)	0.0349 (8)	0.0353 (8)	0.0062 (6)	−0.0017 (6)	−0.0014 (7)
C31	0.0436 (9)	0.0452 (10)	0.0244 (7)	0.0200 (8)	0.0098 (7)	0.0062 (7)
C32	0.0281 (7)	0.0145 (6)	0.0204 (6)	−0.0014 (5)	0.0005 (5)	−0.0013 (5)
C33	0.0233 (6)	0.0160 (6)	0.0225 (6)	0.0010 (5)	0.0006 (5)	−0.0032 (5)
C34	0.0257 (6)	0.0196 (6)	0.0181 (6)	0.0000 (5)	0.0022 (5)	−0.0029 (5)
C35	0.0240 (6)	0.0201 (6)	0.0195 (6)	−0.0005 (5)	−0.0004 (5)	−0.0048 (5)
C36	0.0254 (7)	0.0187 (6)	0.0212 (6)	−0.0044 (5)	0.0022 (5)	−0.0039 (5)
C37	0.0444 (9)	0.0202 (7)	0.0243 (7)	0.0020 (6)	−0.0001 (6)	0.0034 (5)
C38	0.0247 (7)	0.0269 (7)	0.0379 (8)	0.0045 (6)	0.0019 (6)	−0.0025 (6)
C39	0.0353 (8)	0.0356 (8)	0.0208 (7)	0.0014 (7)	0.0071 (6)	0.0002 (6)
C40	0.0289 (7)	0.0373 (9)	0.0278 (7)	0.0042 (6)	−0.0066 (6)	−0.0103 (6)
C41	0.0315 (8)	0.0343 (8)	0.0339 (8)	−0.0104 (6)	0.0085 (6)	−0.0019 (7)
O1	0.0171 (4)	0.0203 (4)	0.0165 (4)	−0.0010 (3)	0.0008 (3)	0.0016 (3)
O2	0.0204 (4)	0.0268 (5)	0.0152 (4)	−0.0009 (4)	0.0015 (3)	0.0021 (4)
Si1	0.02271 (18)	0.0256 (2)	0.02383 (19)	−0.00627 (15)	0.00793 (14)	−0.00108 (15)
Si2	0.01993 (17)	0.0307 (2)	0.01937 (18)	−0.00313 (15)	−0.00191 (14)	−0.00152 (15)
Zr1	0.01765 (6)	0.01484 (6)	0.01414 (6)	−0.00022 (4)	0.00003 (4)	0.00078 (4)
C11A	0.0217 (9)	0.0249 (11)	0.0329 (10)	0.0013 (8)	0.0037 (7)	−0.0032 (9)
C12A	0.0308 (7)	0.0647 (17)	0.0350 (10)	−0.0145 (10)	0.0093 (7)	−0.0062 (10)
C13A	0.0308 (7)	0.0647 (17)	0.0350 (10)	−0.0145 (10)	0.0093 (7)	−0.0062 (10)
C11B	0.0217 (9)	0.0249 (11)	0.0329 (10)	0.0013 (8)	0.0037 (7)	−0.0032 (9)
C12B	0.0308 (7)	0.0647 (17)	0.0350 (10)	−0.0145 (10)	0.0093 (7)	−0.0062 (10)
C13B	0.0308 (7)	0.0647 (17)	0.0350 (10)	−0.0145 (10)	0.0093 (7)	−0.0062 (10)

### Geometric parameters (Å, º)

**Table:**

Al1—O2	1.9016 (10)	C24—C25	1.4257 (19)
Al1—C10	1.9912 (14)	C24—C29	1.507 (2)
Al1—C18	1.9988 (14)	C24—Zr1	2.5348 (13)
Al1—C14	2.0062 (15)	C25—C26	1.4121 (19)
C1—C2	1.3679 (18)	C25—C30	1.501 (2)
C1—Si1	1.8934 (13)	C25—Zr1	2.5466 (13)
C1—Zr1	2.3508 (13)	C26—C31	1.505 (2)
C2—C3	1.5055 (18)	C26—Zr1	2.5354 (14)
C2—Si2	1.9224 (13)	C27—H27A	0.9800
C3—O2	1.2605 (15)	C27—H27B	0.9800
C3—O1	1.2819 (15)	C27—H27C	0.9800
C4—Si1	1.8814 (17)	C28—H28A	0.9800
C4—H4A	0.9800	C28—H28B	0.9800
C4—H4B	0.9800	C28—H28C	0.9800
C4—H4C	0.9800	C29—H29A	0.9800
C5—Si1	1.8863 (18)	C29—H29B	0.9800
C5—H5A	0.9800	C29—H29C	0.9800
C5—H5B	0.9800	C30—H30A	0.9800
C5—H5C	0.9800	C30—H30B	0.9800
C6—Si1	1.8862 (17)	C30—H30C	0.9800
C6—H6A	0.9800	C31—H31A	0.9800
C6—H6B	0.9800	C31—H31B	0.9800
C6—H6C	0.9800	C31—H31C	0.9800
C7—Si2	1.8719 (16)	C32—C36	1.4170 (19)
C7—H7A	0.9800	C32—C33	1.4202 (19)
C7—H7B	0.9800	C32—C37	1.5010 (19)
C7—H7C	0.9800	C32—Zr1	2.5331 (13)
C8—Si2	1.8658 (18)	C33—C34	1.4264 (19)
C8—H8A	0.9800	C33—C38	1.4980 (19)
C8—H8B	0.9800	C33—Zr1	2.5485 (13)
C8—H8C	0.9800	C34—C35	1.4188 (19)
C9—Si2	1.8716 (18)	C34—C39	1.5051 (19)
C9—H9A	0.9800	C34—Zr1	2.5511 (13)
C9—H9B	0.9800	C35—C36	1.4216 (19)
C9—H9C	0.9800	C35—C40	1.5010 (19)
C10—C11B	1.531 (6)	C35—Zr1	2.5571 (13)
C10—C11A	1.536 (2)	C36—C41	1.506 (2)
C10—H10A	0.9900	C36—Zr1	2.5256 (13)
C10—H10B	0.9900	C37—H37A	0.9800
C10—H10C	0.9900	C37—H37B	0.9800
C10—H10D	0.9900	C37—H37C	0.9800
C14—C15	1.524 (2)	C38—H38A	0.9800
C14—H14A	0.9900	C38—H38B	0.9800
C14—H14B	0.9900	C38—H38C	0.9800
C15—C17	1.520 (2)	C39—H39A	0.9800
C15—C16	1.530 (2)	C39—H39B	0.9800
C15—H15	1.0000	C39—H39C	0.9800
C16—H16A	0.9800	C40—H40A	0.9800
C16—H16B	0.9800	C40—H40B	0.9800
C16—H16C	0.9800	C40—H40C	0.9800
C17—H17A	0.9800	C41—H41A	0.9800
C17—H17B	0.9800	C41—H41B	0.9800
C17—H17C	0.9800	C41—H41C	0.9800
C18—C19	1.542 (2)	O1—Zr1	2.0891 (9)
C18—H18A	0.9900	C11A—C13A	1.518 (3)
C18—H18B	0.9900	C11A—C12A	1.534 (5)
C19—C21	1.525 (2)	C11A—H11A	1.0000
C19—C20	1.528 (2)	C12A—H12A	0.9800
C19—H19	1.0000	C12A—H12B	0.9800
C20—H20A	0.9800	C12A—H12C	0.9800
C20—H20B	0.9800	C13A—H13A	0.9800
C20—H20C	0.9800	C13A—H13B	0.9800
C21—H21A	0.9800	C13A—H13C	0.9800
C21—H21B	0.9800	C11B—C13B	1.520 (2)
C21—H21C	0.9800	C11B—C12B	1.525 (14)
C22—C26	1.410 (2)	C11B—H11B	1.0000
C22—C23	1.418 (2)	C12B—H12D	0.9800
C22—C27	1.503 (2)	C12B—H12E	0.9800
C22—Zr1	2.5388 (14)	C12B—H12F	0.9800
C23—C24	1.415 (2)	C13B—H13D	0.9800
C23—C28	1.498 (2)	C13B—H13E	0.9800
C23—Zr1	2.5509 (14)	C13B—H13F	0.9800
			
O2—Al1—C10	107.81 (5)	C36—C32—Zr1	73.44 (8)
O2—Al1—C18	104.24 (5)	C33—C32—Zr1	74.37 (7)
C10—Al1—C18	112.72 (6)	C37—C32—Zr1	121.76 (9)
O2—Al1—C14	101.51 (6)	C32—C33—C34	107.95 (12)
C10—Al1—C14	111.95 (6)	C32—C33—C38	124.70 (13)
C18—Al1—C14	117.21 (6)	C34—C33—C38	126.19 (13)
C2—C1—Si1	122.20 (10)	C32—C33—Zr1	73.17 (7)
C2—C1—Zr1	111.23 (9)	C34—C33—Zr1	73.86 (8)
Si1—C1—Zr1	126.57 (6)	C38—C33—Zr1	128.42 (9)
C1—C2—C3	114.52 (11)	C35—C34—C33	107.67 (12)
C1—C2—Si2	135.55 (10)	C35—C34—C39	124.34 (13)
C3—C2—Si2	109.77 (9)	C33—C34—C39	126.63 (13)
O2—C3—O1	121.03 (12)	C35—C34—Zr1	74.11 (7)
O2—C3—C2	120.17 (11)	C33—C34—Zr1	73.66 (7)
O1—C3—C2	118.79 (11)	C39—C34—Zr1	128.38 (10)
Si1—C4—H4A	109.5	C34—C35—C36	108.27 (12)
Si1—C4—H4B	109.5	C34—C35—C40	124.98 (13)
H4A—C4—H4B	109.5	C36—C35—C40	125.12 (13)
Si1—C4—H4C	109.5	C34—C35—Zr1	73.64 (8)
H4A—C4—H4C	109.5	C36—C35—Zr1	72.54 (7)
H4B—C4—H4C	109.5	C40—C35—Zr1	131.20 (10)
Si1—C5—H5A	109.5	C32—C36—C35	107.90 (12)
Si1—C5—H5B	109.5	C32—C36—C41	126.26 (13)
H5A—C5—H5B	109.5	C35—C36—C41	125.56 (13)
Si1—C5—H5C	109.5	C32—C36—Zr1	74.02 (8)
H5A—C5—H5C	109.5	C35—C36—Zr1	74.98 (8)
H5B—C5—H5C	109.5	C41—C36—Zr1	121.80 (10)
Si1—C6—H6A	109.5	C32—C37—H37A	109.5
Si1—C6—H6B	109.5	C32—C37—H37B	109.5
H6A—C6—H6B	109.5	H37A—C37—H37B	109.5
Si1—C6—H6C	109.5	C32—C37—H37C	109.5
H6A—C6—H6C	109.5	H37A—C37—H37C	109.5
H6B—C6—H6C	109.5	H37B—C37—H37C	109.5
Si2—C7—H7A	109.5	C33—C38—H38A	109.5
Si2—C7—H7B	109.5	C33—C38—H38B	109.5
H7A—C7—H7B	109.5	H38A—C38—H38B	109.5
Si2—C7—H7C	109.5	C33—C38—H38C	109.5
H7A—C7—H7C	109.5	H38A—C38—H38C	109.5
H7B—C7—H7C	109.5	H38B—C38—H38C	109.5
Si2—C8—H8A	109.5	C34—C39—H39A	109.5
Si2—C8—H8B	109.5	C34—C39—H39B	109.5
H8A—C8—H8B	109.5	H39A—C39—H39B	109.5
Si2—C8—H8C	109.5	C34—C39—H39C	109.5
H8A—C8—H8C	109.5	H39A—C39—H39C	109.5
H8B—C8—H8C	109.5	H39B—C39—H39C	109.5
Si2—C9—H9A	109.5	C35—C40—H40A	109.5
Si2—C9—H9B	109.5	C35—C40—H40B	109.5
H9A—C9—H9B	109.5	H40A—C40—H40B	109.5
Si2—C9—H9C	109.5	C35—C40—H40C	109.5
H9A—C9—H9C	109.5	H40A—C40—H40C	109.5
H9B—C9—H9C	109.5	H40B—C40—H40C	109.5
C11B—C10—Al1	120.9 (2)	C36—C41—H41A	109.5
C11A—C10—Al1	117.46 (11)	C36—C41—H41B	109.5
C11A—C10—H10A	107.9	H41A—C41—H41B	109.5
Al1—C10—H10A	107.9	C36—C41—H41C	109.5
C11A—C10—H10B	107.9	H41A—C41—H41C	109.5
Al1—C10—H10B	107.9	H41B—C41—H41C	109.5
H10A—C10—H10B	107.2	C3—O1—Zr1	120.99 (8)
C11B—C10—H10C	107.1	C3—O2—Al1	134.62 (9)
Al1—C10—H10C	107.1	C4—Si1—C6	105.39 (8)
C11B—C10—H10D	107.1	C4—Si1—C5	106.32 (9)
Al1—C10—H10D	107.1	C6—Si1—C5	110.14 (8)
H10C—C10—H10D	106.8	C4—Si1—C1	110.82 (7)
C15—C14—Al1	121.07 (10)	C6—Si1—C1	112.36 (7)
C15—C14—H14A	107.1	C5—Si1—C1	111.48 (7)
Al1—C14—H14A	107.1	C8—Si2—C9	111.40 (9)
C15—C14—H14B	107.1	C8—Si2—C7	104.85 (8)
Al1—C14—H14B	107.1	C9—Si2—C7	104.33 (9)
H14A—C14—H14B	106.8	C8—Si2—C2	110.57 (7)
C17—C15—C14	111.16 (13)	C9—Si2—C2	106.56 (7)
C17—C15—C16	109.32 (14)	C7—Si2—C2	119.00 (7)
C14—C15—C16	112.22 (13)	O1—Zr1—C1	74.44 (4)
C17—C15—H15	108.0	O1—Zr1—C36	85.03 (4)
C14—C15—H15	108.0	C1—Zr1—C36	125.20 (4)
C16—C15—H15	108.0	O1—Zr1—C32	76.58 (4)
C15—C16—H16A	109.5	C1—Zr1—C32	92.80 (4)
C15—C16—H16B	109.5	C36—Zr1—C32	32.53 (4)
H16A—C16—H16B	109.5	O1—Zr1—C24	129.72 (4)
C15—C16—H16C	109.5	C1—Zr1—C24	117.27 (5)
H16A—C16—H16C	109.5	C36—Zr1—C24	114.94 (5)
H16B—C16—H16C	109.5	C32—Zr1—C24	143.03 (5)
C15—C17—H17A	109.5	O1—Zr1—C26	76.27 (4)
C15—C17—H17B	109.5	C1—Zr1—C26	113.37 (5)
H17A—C17—H17B	109.5	C36—Zr1—C26	109.85 (5)
C15—C17—H17C	109.5	C32—Zr1—C26	134.96 (5)
H17A—C17—H17C	109.5	C24—Zr1—C26	53.82 (4)
H17B—C17—H17C	109.5	O1—Zr1—C22	81.45 (4)
C19—C18—Al1	116.89 (9)	C1—Zr1—C22	84.83 (5)
C19—C18—H18A	108.1	C36—Zr1—C22	141.96 (5)
Al1—C18—H18A	108.1	C32—Zr1—C22	157.71 (5)
C19—C18—H18B	108.1	C24—Zr1—C22	53.86 (5)
Al1—C18—H18B	108.1	C26—Zr1—C22	32.27 (5)
H18A—C18—H18B	107.3	O1—Zr1—C25	104.17 (4)
C21—C19—C20	109.70 (13)	C1—Zr1—C25	137.36 (4)
C21—C19—C18	111.31 (12)	C36—Zr1—C25	96.52 (5)
C20—C19—C18	112.88 (13)	C32—Zr1—C25	128.98 (5)
C21—C19—H19	107.6	C24—Zr1—C25	32.59 (4)
C20—C19—H19	107.6	C26—Zr1—C25	32.26 (4)
C18—C19—H19	107.6	C22—Zr1—C25	53.53 (5)
C19—C20—H20A	109.5	O1—Zr1—C33	102.80 (4)
C19—C20—H20B	109.5	C1—Zr1—C33	81.73 (4)
H20A—C20—H20B	109.5	C36—Zr1—C33	53.85 (4)
C19—C20—H20C	109.5	C32—Zr1—C33	32.46 (4)
H20A—C20—H20C	109.5	C24—Zr1—C33	126.62 (5)
H20B—C20—H20C	109.5	C26—Zr1—C33	163.45 (5)
C19—C21—H21A	109.5	C22—Zr1—C33	164.18 (5)
C19—C21—H21B	109.5	C25—Zr1—C33	137.37 (4)
H21A—C21—H21B	109.5	O1—Zr1—C23	113.05 (5)
C19—C21—H21C	109.5	C1—Zr1—C23	87.17 (5)
H21A—C21—H21C	109.5	C36—Zr1—C23	146.98 (5)
H21B—C21—H21C	109.5	C32—Zr1—C23	169.89 (5)
C26—C22—C23	107.76 (13)	C24—Zr1—C23	32.32 (5)
C26—C22—C27	124.71 (16)	C26—Zr1—C23	53.37 (5)
C23—C22—C27	127.10 (16)	C22—Zr1—C23	32.34 (5)
C26—C22—Zr1	73.73 (8)	C25—Zr1—C23	53.38 (5)
C23—C22—Zr1	74.30 (8)	C33—Zr1—C23	137.91 (5)
C27—C22—Zr1	123.70 (10)	O1—Zr1—C34	130.43 (4)
C24—C23—C22	108.41 (13)	C1—Zr1—C34	105.24 (4)
C24—C23—C28	125.21 (16)	C36—Zr1—C34	53.92 (4)
C22—C23—C28	125.21 (17)	C32—Zr1—C34	53.85 (4)
C24—C23—Zr1	73.21 (8)	C24—Zr1—C34	95.39 (5)
C22—C23—Zr1	73.36 (8)	C26—Zr1—C34	138.49 (5)
C28—C23—Zr1	129.12 (11)	C22—Zr1—C34	147.93 (5)
C23—C24—C25	107.40 (13)	C25—Zr1—C34	106.71 (4)
C23—C24—C29	124.92 (14)	C33—Zr1—C34	32.49 (4)
C25—C24—C29	126.72 (14)	C23—Zr1—C34	116.45 (5)
C23—C24—Zr1	74.47 (8)	O1—Zr1—C35	117.26 (4)
C25—C24—Zr1	74.16 (8)	C1—Zr1—C35	134.79 (4)
C29—C24—Zr1	125.84 (10)	C36—Zr1—C35	32.48 (4)
C26—C25—C24	107.93 (13)	C32—Zr1—C35	53.60 (4)
C26—C25—C30	123.43 (13)	C24—Zr1—C35	89.44 (5)
C24—C25—C30	127.38 (13)	C26—Zr1—C35	111.84 (5)
C26—C25—Zr1	73.43 (8)	C22—Zr1—C35	138.06 (5)
C24—C25—Zr1	73.25 (8)	C25—Zr1—C35	84.76 (4)
C30—C25—Zr1	129.13 (10)	C33—Zr1—C35	53.47 (4)
C22—C26—C25	108.47 (13)	C23—Zr1—C35	120.57 (5)
C22—C26—C31	126.77 (14)	C34—Zr1—C35	32.25 (4)
C25—C26—C31	124.58 (14)	C13A—C11A—C12A	110.4 (2)
C22—C26—Zr1	74.00 (8)	C13A—C11A—C10	112.23 (18)
C25—C26—Zr1	74.31 (8)	C12A—C11A—C10	110.0 (2)
C31—C26—Zr1	121.66 (10)	C13A—C11A—H11A	108.0
C22—C27—H27A	109.5	C12A—C11A—H11A	108.1
C22—C27—H27B	109.5	C10—C11A—H11A	108.0
H27A—C27—H27B	109.5	C11A—C12A—H12A	109.5
C22—C27—H27C	109.5	C11A—C12A—H12B	109.5
H27A—C27—H27C	109.5	H12A—C12A—H12B	109.5
H27B—C27—H27C	109.5	C11A—C12A—H12C	109.5
C23—C28—H28A	109.5	H12A—C12A—H12C	109.5
C23—C28—H28B	109.5	H12B—C12A—H12C	109.5
H28A—C28—H28B	109.5	C11A—C13A—H13A	109.5
C23—C28—H28C	109.5	C11A—C13A—H13B	109.5
H28A—C28—H28C	109.5	H13A—C13A—H13B	109.5
H28B—C28—H28C	109.5	C11A—C13A—H13C	109.5
C24—C29—H29A	109.5	H13A—C13A—H13C	109.5
C24—C29—H29B	109.5	H13B—C13A—H13C	109.5
H29A—C29—H29B	109.5	C13B—C11B—C12B	104.5 (7)
C24—C29—H29C	109.5	C13B—C11B—C10	111.9 (5)
H29A—C29—H29C	109.5	C12B—C11B—C10	115.5 (5)
H29B—C29—H29C	109.5	C13B—C11B—H11B	108.2
C25—C30—H30A	109.5	C12B—C11B—H11B	108.2
C25—C30—H30B	109.5	C10—C11B—H11B	108.2
H30A—C30—H30B	109.5	C11B—C12B—H12D	109.5
C25—C30—H30C	109.5	C11B—C12B—H12E	109.5
H30A—C30—H30C	109.5	H12D—C12B—H12E	109.5
H30B—C30—H30C	109.5	C11B—C12B—H12F	109.5
C26—C31—H31A	109.5	H12D—C12B—H12F	109.5
C26—C31—H31B	109.5	H12E—C12B—H12F	109.5
H31A—C31—H31B	109.5	C11B—C13B—H13D	109.5
C26—C31—H31C	109.5	C11B—C13B—H13E	109.5
H31A—C31—H31C	109.5	H13D—C13B—H13E	109.5
H31B—C31—H31C	109.5	C11B—C13B—H13F	109.5
C36—C32—C33	108.17 (12)	H13D—C13B—H13F	109.5
C36—C32—C37	126.46 (13)	H13E—C13B—H13F	109.5
C33—C32—C37	125.20 (13)		
